# Pattern of Changes in Age-Specific Fertility Rates, Total Fertility Rate, and Cohort Fertility Rate in Rural Areas of Fars Province, Southern Iran (1988-2012) 

**Published:** 2019-03

**Authors:** Haleh Ghaem, Marjan Zare, Abdulrasool Hemmati, Mohsen Moghadami, Fariba Moradi, Ali Semati

**Affiliations:** 1 Department of Epidemiology, Research Center for Health Sciences, School of Health, Shiraz University of Medical Sciences, Shiraz, Iran; 2 Department of Epidemiology, School of Health, Shiraz University of Medical Sciences, Shiraz, Iran; 3 Vice Chancellor of Health Affairs, Non-Communicable Diseases Research Center, Shiraz University of Medical Sciences, Shiraz, Iran; 4 Department of Medicine, Research Center for HIV/AIDS, Shiraz University of Medical Sciences, Shiraz, Iran; 5 Department of Social Medicine, Family and Population Health Administration, Deputy of Health, Shiraz University of Medical Sciences, Shiraz, Iran; 6 Vice Chancellor of Health, Shiraz University of Medical Sciences, Shiraz, Iran

**Keywords:** Age-Specific Fertility Rate (ASFR), Total Fertility Rate (TFR), Cohort Fertility Rate (CFR)

## Abstract

**Objective:** To investigate the trend of changes in Age-Specific Fertility Rate (ASFR), Total Fertility Rate (TFR), and Cohort Fertility Rate (CFR) in rural areas of Fars province, southern Iran during 1988-2012.

**Materials and methods:** This cross-sectional study was conducted based on analyze fluctuations in fertility. Information about the number of births and mothers aged 15-49 years was collected. By calculating the ASFR, TFR, and CFR along with analyzing their patterns the trend of changes in fertility rate would be revealed. Finally, modeling and time series forecast of ASFR based on age groups was conducted using the SPSS software.

**Results:** The TFR was estimated to be 4.21, 2.1, 1.76, 1.65, and 1.78 per thousand in 1992, 1997, 2002, 2007, and 2012, respectively. Moreover, CFR was 2.01, 1.54, 1.05, 0.54, and 0.13 for those born during 1972-1976, 1977-1981, 1982-1986, 1987-1990, and 1991-1995, respectively. Also the time trend of ASFR based on age groups showed a negative slope.

**Conclusion:** The fertility followed a negative slope during 1992-2012, indicating their descending trend during these years. TFR = 2.1 was a standard population replacement rate in the societies. Therefore, continual decline of this rate during 1992-2012 could be a warning factor that requires planning for reform and precise evaluation.

## Introduction

Fertility, mortality, and migration affect population changes. In the absence of migration, reproduction is the only way to compensate for death and if fertility is greater than mortality, population growth will be positive. In the middle of the 19^th^ century, the mean number deliveries was 6 children per woman in the U.S. and Australia, while currently the mean number of deliveries is less than 2 children per woman in these countries (1). In general, fertility can be investigated by two methods namely, periodical and cohort. The main characteristic in periodical analysis of fertility is its segmental perspective; i.e., births should be analyzed annually. On the other hand, fertility is calculated vertically in the cohort model. This implies that births are evaluated in a special group of women, usually those with the same date of birth (2). Marriage and women’s level of education respectively have the most positive and the most negative effects on the fertility rate. Then, unemployment, family planning policies, policies of paying cash subsidies and total annual household expenses have reverse effects on the fertility rate and the policies of paying cash subsidies and Sunni population have positive effects on the fertility rate (3). Iran experienced TFR below replacement level from 2006 (1.85). Results of next census (2011) revealed more decline in the country TFR (1.8) and it has been continued to be under replacement level. Age of marriage among Iranian girls and boys is in the increasing trend and it has been increased to 23.4 for girls and above 27 for boys (4). Cohort is a new method for assessment of fertility rate, which has been applied since 30 years ago. It is in fact a method for analyzing the trends and the levels of fertility rather than its prediction. Compared to managers and planners, academic researchers are more interested in this kind of procedure (2).

Considering the important role of fertility as an essential basis in the society and the continual warning about the descending trend of fertility, we aimed to investigate the trend of changes in age-specific fertility rate (ASFR), total fertility rate (TFR), and Cohort fertility rate (CFR) in Fars Province, southern Iran.

***Data source: ***The data used in the analysis of fertility trend were extracted from the vital horoscope in the rural health centers of Fars province. The data included the number of mothers and live births in the rural areas during 1988-2012.

## Materials and methods

This study was approved by Ethic committee of Shiraz University of Medical Sciences and consent has been obtained from patients. In this cross-sectional study, data were collected about all births of the women aged 15-49 years in rural areas of Fars province, south of Iran during 1988-2012 from the vital horoscope in rural health center, in order to investigate ASFR, TFR, and CFR trends data were calculated according to the following formula and were entered into tables and graphs as ASFR, TFR, and CFR.

$\mathrm{CFR}=\frac{\sum \mathrm{ASFR*5}}{1000}$

$\mathrm{TFR}=\frac{\sum \mathrm{ASFR*5}}{1000}$

$\mathrm{ASFR}=\frac{\mathrm{number of birth}}{\mathrm{female popuion}}\mathrm{*1000}$

CFR can be presented as the ultimate dimension of a family. In other words, it is the mean number of children that a women could give birth to in her fertility period. CFR studies are conducted during a specific period of time and are used for describing the trend and level of fertility rather than anticipating it (2). 

Then, using SPSS software performed time series forecast of ASFR based on age groups from 1988 to 2018.

In this study, urban population of the women aged 15-49 years, women under 15 and above 49 years of age, stillbirth, and miscarriage were not recorded.

## Results

Based on the results of this study, ASFR in the rural areas of Fars province, southern Iran during 1992-2012 has been presented in specific age scales displayed column by column in 5-year sequences based on age categories. Nonetheless, this columnar frame just reveals the cross-effect of the aforementioned scales.TFR was calculated as 4.21, 2.1, 1.76, 1.65, and 1.78 per thousand in 1992, 1997, 2002, 2007, and 2012, respectively (Table 1).

In order to assess the role of cohort effects as an important variable in this study (ASFR in cohort births), considering the diagonals of table 1, fertility can be observed in cohort groups. Therefore, each ASFR can be related to one special age group, one special period of time, and one special cohort birth. CFR could also be calculated based on table 2. Using the total rows of table 2 in CFR formula, it was computed as 2.01, 1.56, 1.05, 0.54, and 0.13 for the women born during 1972-1976, 1977-1981, 1982-1986, 1987-1990, and 1991-1995, respectively. 

**Table 1:** ASFR and TFR in rural areas of Fars provinces, Iran

**Year**	**1992**	**1997**	**2002**	**2007**	**2012**
Age Group	ASFR	ASFR	ASFR	ASFR	ASFR
15-19	69	35	25	23	25
20-24	180	112	89	83	84
25-29	198	117	104	100	101
30-34	172	87	82	69	85
35-39	133	46	40	44	48
40-44	70	19	11	11	13
45-49	21	4	2	1	1
TFR	4.21	2.1	1.76	1.65	1.78

Also, in order to accurately evaluate the age-specific fertility rate (ASFR) in seven age groups of women: 15-19, 20-24, 25-29, 30-34, 35-39, 40-44, 45-49 years old, Modeling and time prediction were performed and the results showed the trend of ASFR changed from 1988 to 2018.

**Figure 1:**
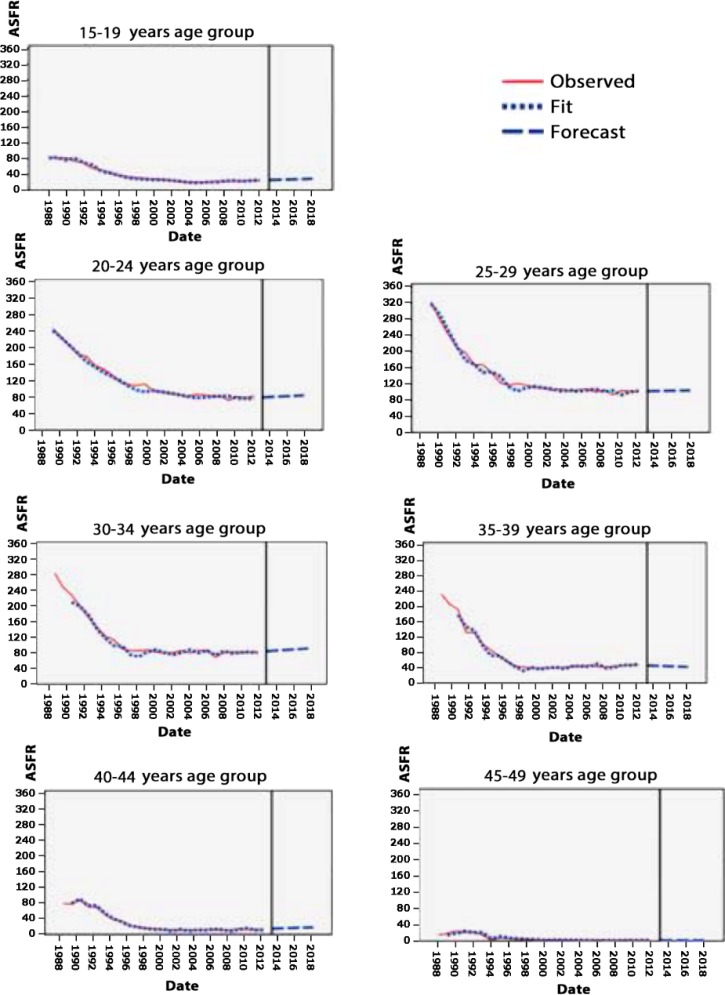
Modeling and prediction of ASFR based on age groups from 1988 to 2018

According to modeling (Figure 1), the highest ASFR was related to the women aged 20-24, 25-29, 30-34, and 35-39 years in 1988. However, in 2012 this trend decreased and got closer to other age groups. Analyzing the graphs in results showed that for the women aged 15-19 years, ASFR was 86 per 1000 women at the beginning of 1988, this measure decreased to 25 in 2012, and it has been forecasted to reach 35 by 2018. For the individuals aged 20-24 years, ASFR was 248 per 1000 women at the beginning of 1988, which decreased to 84 in 2012 and it has been predicted to reach 91 by 2018. In the individuals aged 25-29 years, ASFR was 324 per 1000 women at the beginning of 1988, which reduced to 101 in 2012 and it has been forecasted to reach 108 by 2018. Besides, this measure was 287 per 1000 women aged 30-34 years at the beginning of 1988, which decreased to 85 in 2012 and it has been forecasted to reach 96 by 2018. In the individuals aged 35-39 years, ASFR was 234 per 1000 women at the beginning of 1988, which decreased to 48 in 2012 and it has been forecasted to reach 38 by 2018. In the individuals aged 40-44 years also, ASFR was 79 per 1000 women at the beginning of 1988, which reduced to 13 in 2012 and it has been forecasted to reach 20 by 2018. Finally, ASFR was 15 per 1000 women for the individuals aged 45-49 years at the beginning of 1988, this measure decreased to 1 in 2012 and it has been predicted to remain 1 by 2018.

## Discussion

The results of this study indicated that TFR and CFR had significant reductions and in this scale, the negative slope revealed the descending trend of fertility in the rural areas of Fars province, southern Iran during 1988-2012. TFR reduced from 4.21 in 1992 to 1.78 in 2012 and Considering TFR=2.1 as a standard figure, continual decline of this rate could be a warning factor requiring precise evaluation. The time trend of ASFR based on age groups during 1988-2012 showed the negative slope of trend of ASFR during the aforementioned years.

Women’s mean age of first-time pregnancy increased in US from 21 to 25 years in the 40 years after 1970, with a decrease of mothers younger than 20 years of age, and a sensible increase of those older than 35 (5).Relationship between the age of the female partner and fertility showed that: by age 30, 7% of couples were infertile, by age 35, 11% of couples were infertile, and by age 40 and 45, 33% and 87% of couples were infertile, respectively (6). Significant correlations exist between mothers’ age, occupation and quality of healthcare and their level of health literacy (p ˂ 0.05) (7). There is a negative relationship between problematic confrontation and infertility stress as well as positive relationship between exciting confrontation and infertility stress (8). Wide knowledge gap was found between Bangladeshi urban and rural respondents regarding their reproductive behaviors.

**Table 2:** ASFRs in cohort births and CFR of rural areas of Fars province

**Year of Birth**	**Age group**							**CFR**
	**15-19**	**20-24**	**25-29**	**30-34**	**35-39**	**40-44**	**45-49**	
1972-76	69	112	104	69	48			2.01
1977-81	35	89	100	85				1.54
1982-86	25	83	101					1.05
1987-90	23	84						0.54
1991-95	25							0.13

(Time series modeling)

Government and concerned organizations should promote and strengthen various health education programs to focus on reproductive health, especially among reproductive aged women in rural area (9). In the East Ethiopia, only 39.3% female adolescents have ever used family planning, and 45.8% adolescents have ever used voluntary counseling and testing services. Lack of adolescent reproductive health services, harmful traditional practices, lack of privacy and inconvenient service hour were reasons for not utilizing the service (10). Relationship between unemployment and total fertility showed that, although fertility was counter-cyclical before 1970, with good economic times being associated with lower fertility, since then it has become pro-cyclical, with good economic times being associated with higher fertility (11). Lack of proper access to health services has been associated with serious ill-health ranging from reproductive tract infection, urinary tract infection, etc. Also significant association was observed between having good/fair knowledge and good practices about reproductive health (12). Male unemployment is related to a postponement of first and second childbearing in both Germany and Denmark. The role of female unemployment is less clear at these two parities. Both male and female unemployment is positively correlated with third birth risks. (13). Aging has severe effect on men’s and women’s reproductive health. It is predicted elderly explosion will occur in Iran in 1410 and 25 – 30 percent of population will above 50 years old (14). It is predicted that Japan and European Union will soon experience appreciable decreases in their populations due to persistently low total fertility rates (TFR) below replacement level (2.1 child per woman). In the United States, where TFR has also declined, there are ethnic differences. Caucasians have rates below replacement, while TFRs among African-Americans and Hispanics are higher (15). International investigation into the relationship between social expenditure for family and total fertility rate showed that, In the correlation analysis for total fertility rates and family-related social spending to gross domestic product (GDP) ratio, the benefits-in-kind to GDP ratio and total fertility rates indicated a trend toward correlation (r = 0.32, p = 0.06). In addition, the results for the partial correlation between family-related social spending to GDP ratio and total fertility rates showed a significant correlation between the two (16). Comparison in two groups of people for decompose differences in total fertility rate showed that, socioeconomic deprivation, Marriage at the age of 15-29, and Low education level have increasing fertility, and those factors are potentially sign of increasing reproductive (17). After reaching the world's population of 2.5 billion in 1950, it grew rapidly to 7.2 billion in 2013 and the projections expect this total to be 10.9 billion by 2100. World regions differ widely in their demographic trends, with rapid population growth and high fertility continuing in the poorest countries, particularly in sub-Saharan Africa, while population decline, population aging, and very low fertility are now a key concern in many developed countries (18).Population structure was aging fast, fertility rates continued to decrease to a substantially low level, and evidence displayed notable socioeconomic issues associated with low-fertility trap (19). The fertility rate was negatively associated with increased outdoor ambient fine particle (PM2.5) concentration levels in the contiguous US from 2003 to 2011. The fertility rate reduction was more strongly linked to PM 2.5 exposure just before pregnancy than that during pregnancy (20).

***Limitation: ***There were some limitations in the present study. Low Records and incorrect registration of women aged 15-49 years and born in vital horoscope, and lack of recording data electronically in the first few years of the study were among the limitations we had.

## Conclusion

The findings of this study showed that the negative slope of TFR, CFR, and ASFR in the rural population of Fars province during 1988-2012, can be considered as a warning factor in population growth rates. Therefore, accurate planning for demographic policies can be an effective step in positive population growth.
